# Screen-identified selective inhibitor of lysine demethylase 5A blocks cancer cell growth and drug resistance

**DOI:** 10.18632/oncotarget.9539

**Published:** 2016-05-21

**Authors:** Molly Gale, Joyce Sayegh, Jian Cao, Michael Norcia, Peter Gareiss, Denton Hoyer, Jane S. Merkel, Qin Yan

**Affiliations:** ^1^ Department of Pathology, Yale School of Medicine, New Haven, CT, USA; ^2^ Yale Center for Molecular Discovery, Yale University, West Haven, CT, USA; ^3^ Current address: Department of Biology and Chemistry, Azusa Pacific University, Azusa, CA, USA

**Keywords:** histone demethylase inhibitor, KDM5A, anti-cancer drug, drug resistance, epigenetics

## Abstract

Lysine demethylase 5A (KDM5A/RBP2/JARID1A) is a histone lysine demethylase that is overexpressed in several human cancers including lung, gastric, breast and liver cancers. It plays key roles in important cancer processes including tumorigenesis, metastasis, and drug tolerance, making it a potential cancer therapeutic target. Chemical tools to analyze KDM5A demethylase activity are extremely limited as available inhibitors are not specific for KDM5A. Here, we characterized KDM5A using a homogeneous luminescence-based assay and conducted a screen of about 9,000 small molecules for inhibitors. From this screen, we identified several 3-thio-1,2,4-triazole compounds that inhibited KDM5A with low μM *in vitro* IC_50_ values. Importantly, these compounds showed great specificity and did not inhibit its close homologue KDM5B (PLU1/JARID1B) or the related H3K27 demethylases KDM6A (UTX) and KDM6B (JMJD3). One compound, named YUKA1, was able to increase H3K4me3 levels in human cells and selectively inhibit the proliferation of cancer cells whose growth depends on KDM5A. As KDM5A was shown to mediate drug tolerance, we investigated the ability of YUKA1 to prevent drug tolerance in EGFR-mutant lung cancer cells treated with gefitinib and HER2+ breast cancer cells treated with trastuzumab. Remarkably, this compound hindered the emergence of drug-tolerant cells, highlighting the critical role of KDM5A demethylase activity in drug resistance. The small molecules presented here are excellent tool compounds for further study of KDM5A's demethylase activity and its contributions to cancer.

## INTRODUCTION

Epigenetic regulators read, write, and erase post-translational modifications of nucleic acids in DNA and amino acids in histone tails. These marks, such as methyl or acetyl groups, are important in the processes of DNA repair and replication and can function to dynamically regulate gene expression [1]. For example, near transcriptional start sites, lysine acetylation and methylation of histone 3 lysine 4 (H3K4) are generally associated with active gene transcription [2]. Deregulation of epigenetic regulators is associated with many human diseases including inflammatory disorders, metabolic disorders, neurological disorders, and cancer [3]. Small molecule inhibitors targeting regulators of the epigenome are becoming increasingly sought after for research and clinical uses. In fact, several histone deacetylase inhibitors and DNA methyltransferase inhibitors were approved for therapeutic use, and many more epigenetic drugs are in clinical trials [4].

Here, we focus on the development of inhibitors of lysine demethylase 5A (KDM5A/RBP2/JARID1A). KDM5A belongs to the KDM5 or JARID1 family of demethylases, which also includes KDM5B (PLU1/JARID1B), KDM5C (SMCX/JARID1C), and KDM5D (SMCY/JARID1D) (for review of KDM5 family proteins, please see [5]). All family members are Jumonji C (JmjC) domain-containing enzymes that demethylate di- and tri-methylated lysine 4 on histone H3 through a dioxygenase reaction requiring cofactors Fe(II) and α-ketoglutarate (α-KG) [6, 7]. KDM5 enzymes contain several conserved domains including Jumonji N, JmjC, AT-rich interactive domain (ARID), and two or three plant homeodomains (PHD). High similarity in domain organization and homology in amino acid sequence is observed between members of the pairs KDM5A/KDM5B and KDM5C/KDM5D. KDM5A is often classified as a transcriptional repressor as it removes methyl groups from H3K4me2 and H3K4me3, marks associated with active promoters [8]. It has been shown to interact with the Sin3 corepressor complex [9] and RBP-J, a repressor of Notch target genes [10]. However, the mono-methylated product of its demethylation reaction is associated with active enhancers. Thus, KDM5A may act as an activator by enriching H3K4me1 at enhancer regions, similar to KDM5C [11].

KDM5A was originally discovered as a binding partner for the retinoblastoma protein (pRB) [12] and subsequently shown to antagonize the function of pRB in differentiation and senescence control [13, 14]. It is amplified in breast cancer [15] and overexpressed in multiple human cancer types, including lung [16, 17], gastric [18, 19] and hepatocellular [20] cancers. KDM5A contributes to several key steps of cancer progression including tumorigenesis, metastasis and drug tolerance and is an attractive therapeutic target (reviewed in [21]). Knockout of KDM5A significantly slowed tumorigenesis in three different genetically engineered mouse models of cancer [14, 22]. For instance, KDM5A knockout drastically increased lifespan in *Rb1*^+/−^ mice, which developed pituitary and thyroid tumors [14]. Similarly, KDM5A loss prolonged survival in mice with loss of multiple endocrine neoplasia type 1 (MEN1) in their pancreatic islet cells, which developed neuroendocrine tumors [14]. In the *MMTV-Neu* breast cancer mouse model, loss of KDM5A slowed tumorigenesis as well as metastasis to the lungs [22]. Similarly, KDM5A was found to be important for epithelial-mesenchymal transition and invasion of lung cancer cells [16, 17]. Furthermore, KDM5A expression is implicated in drug resistance to targeted anti-cancer therapies in both lung [23] and breast cancer [15], as well as in resistance to a DNA alkylating agent in glioblastoma [24].

While there are several compounds that can inhibit the demethylase activity of KDM5A (for example [25–29]), there are currently no specific inhibitors shown to target KDM5A without inhibiting other members of the KDM5 family. Here we describe a screen in a high-throughput screening format and identify small molecule inhibitors of full-length KDM5A. Several 3-thio-1,2,4-triazole compounds we identified inhibit KDM5A, but not KDM5B, KDM6A or KDM6B. One such compound, YUKA1, is cell permeable and selectively attenuates proliferation of several cancer cell lines. Moreover, YUKA1 impedes the outgrowth of cancer cells resistant to targeted anti-cancer therapies, demonstrating the importance of KDM5A demethylase activity in drug resistance and supporting KDM5A inhibition as a potential therapeutic strategy to prevent tumor recurrence.

## RESULTS

### Biochemical characterization of KDM5A

**Figure 1:**
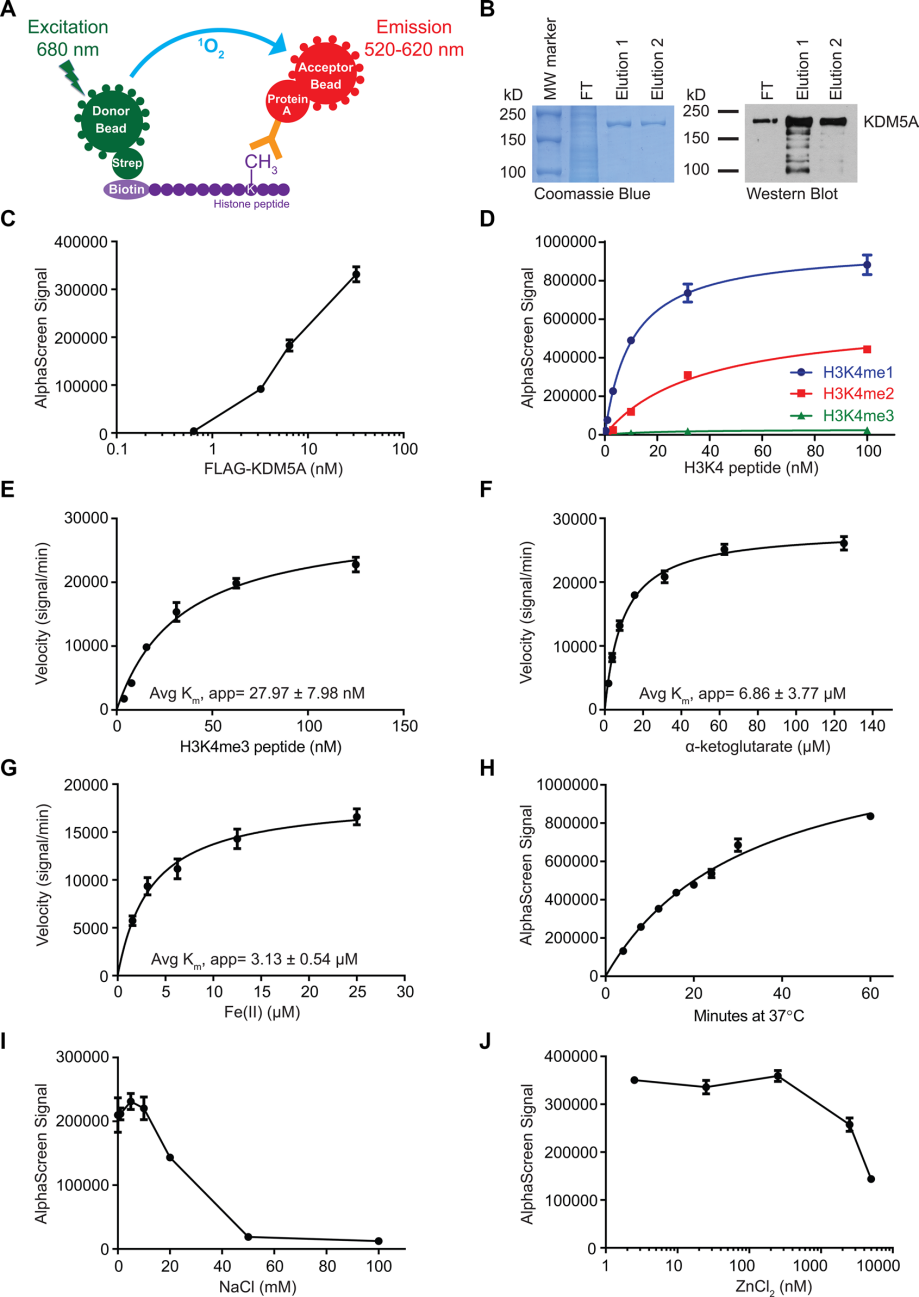
Biochemical characterization of KDM5A using AlphaScreen (**A**) Schematic of the AlphaScreen assay used to measure demethylation of biotinylated H3K4me3 peptides by KDM5A. strep, streptavidin. (**B**) Verification of affinity purified full-length FLAG-KDM5A by Coomassie Brilliant Blue stain (left) and anti-KDM5A western blot (right). MW, molecular weight; FT, flow-through. (**C**) Titration of FLAG-KDM5A in AlphaScreen assays. (**D**) Assessment of the specificity of the H3K4me1/2 antibody using mono-, di-, and tri-methylated H3K4 peptides. (**E**–**G**) Determination of the average apparent K_m_ of H3K4me3 peptide (E), α-KG (F), and Fe(II) (G) from two independent experiments. (**H**) Time course of the KDM5A demethylation reaction. (**I**–**J**) Titration of NaCl (I) and ZnCl_2_ (J) in the KDM5A demethylation reaction. Data points in C-J represent mean ± SD. Data are representative of at least two independent experiments performed in triplicate.

AlphaScreen technology (PerkinElmer) was utilized to perform a screen for small molecule inhibitors of KDM5A. The assay was comprised of two steps, a demethylation reaction followed by detection of the product. A biotinylated H3K4me3 peptide was used as substrate in the demethylation reaction with KDM5A in the presence or absence of small molecule inhibitors. The presence of peptide product (H3K4me1/2) was detected using a product-specific antibody and beads. For this, acceptor beads coated in protein A bound to the antibody, which recognized the peptide product. Donor beads coated in streptavidin bound biotin on the peptide substrate. If the demethylation reaction occurred, the beads were in very close proximity and laser excitation of the donor beads at 680nm caused a transfer of energy in the form of reactive singlet oxygen, resulting in emission by the acceptor beads between 520–620 nm ([30, 31], Figure 1A). The luminescent signal detected was a proxy for the amount of demethylation that occurred.

FLAG-tagged full-length KDM5A was expressed in Sf21 insect cells and affinity purified using the FLAG tag. The purity of the isolated enzyme was assessed by SDS-PAGE and western blot (Figure 1B). The enzyme showed strong activity by AlphaScreen even at low nM concentration (Figure 1C). We selected an antibody with an affinity for H3K4me1 that is about twice its affinity for H3K4me2, enabling detection of not only the incidence of demethylation, but also the degree of demethylation (Figure 1D). The affinity of the enzyme for the peptide in this assay was assessed by measuring the rate of the demethylation reaction over increasing peptide concentrations, leading to an average apparent K_m_ of about 28 nM (Figure 1E). The average apparent K_m_ of α-KG was about 7 μM (Figure 1F). Determination of the reaction rate over a range of Fe(II) concentrations revealed an average apparent K_m_ of about 3 μM (Figure 1G). Under standard conditions, demethylation by FLAG-KDM5A increased linearly up to about 30 minutes, and continued to increase at a slower rate up to one hour (Figure 1H). FLAG-KDM5A was sensitive to high salt concentrations, as the enzyme showed little activity with more than 50 mM NaCl (Figure 1I). It was also sensitive to ZnCl_2_ concentrations above 2 μM (Figure 1J).

### Identification of specific KDM5A inhibitors by screening

**Figure 2:**
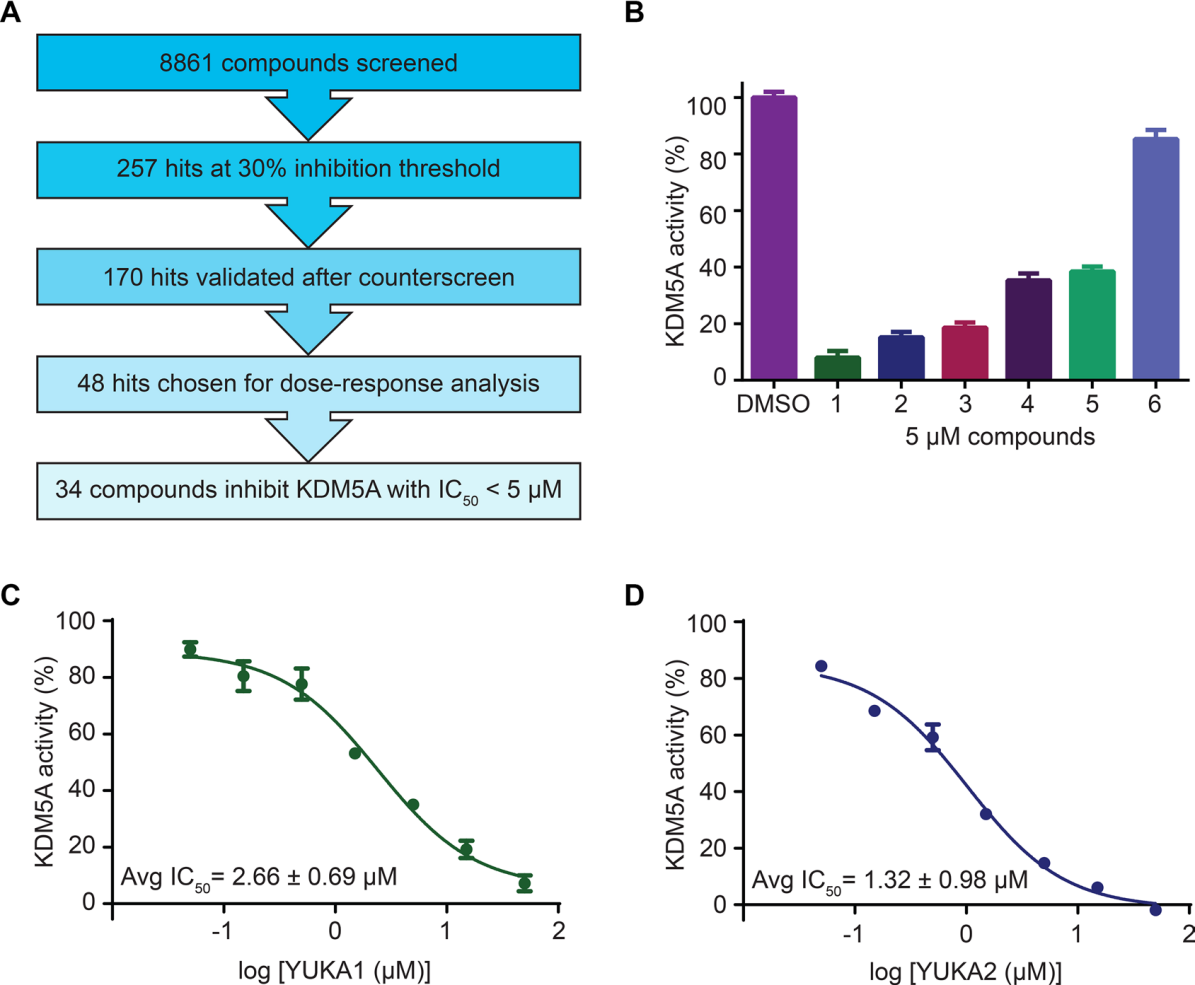
Screen overview and top hits (**A**) Overview of the screening and hit selection process. (**B**) Validation of selected hits with a 3-thio-1,2,4-triazole core. The names and structures of these compounds are listed in Table 1. (**C**–**D**) Dose-response analysis for YUKA1 (C) and YUKA2 (D). Data are representative of at least four independent experiments performed in triplicate. Data points and bars in B-D indicate mean ± SEM.

The screen (see schematic in Figure 2A) included 8,861 compounds from selected small molecule libraries, including drugs approved for use in the clinic and diverse collections of compounds representing broad pharmacophore diversity and bioavailability. Screening statistics showed that the assay was sensitive and robust with a high average signal to background ratio (~12) and an excellent average Z' score (0.75). An inhibition threshold of three standard deviations (about 30% inhibition) identified 257 compounds. A counter-screen was used to eliminate any compounds that interfered with the assay itself by detecting the luminescent signal in the presence of the compound and positive control H3K4me2 peptide. The counter-screen validated 170 compounds. We chose 48 compounds from this list for dose-response analysis, including 42 of the top 44 compounds with the highest potency at 20 μM and 6 compounds with drug-like structures. Among these 48 compounds, 34 compounds inhibited KDM5A *in vitro* with half-maximal inhibitory concentrations (IC_50_) of less than 5 μM.

**Figure 3:**
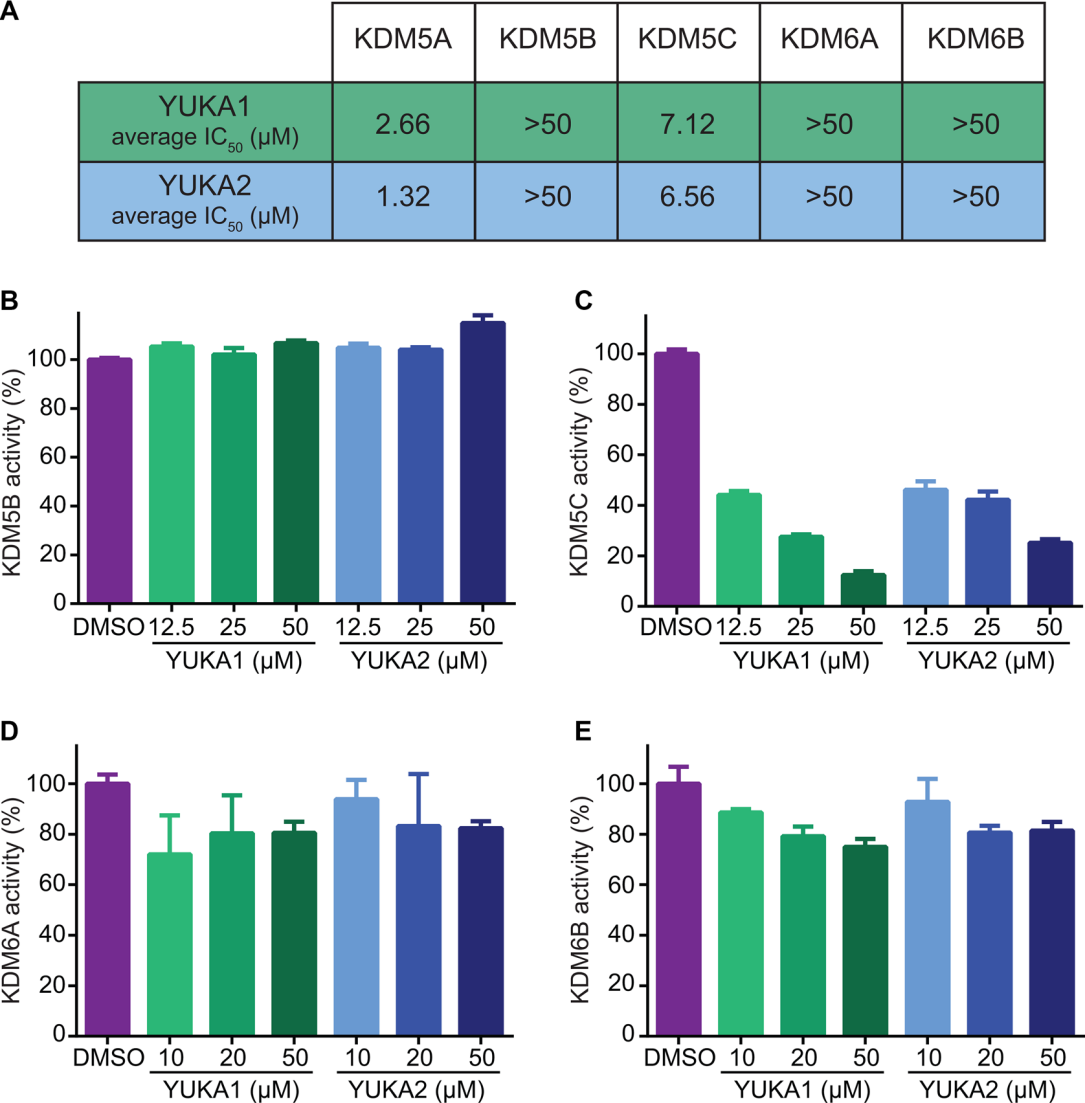
YUKA1 and YUKA2 are KDM5A/C specific inhibitors (**A**) The average IC_50_ values of YUKA1 and YUKA2 for members of the KDM5 and KDM6 families determined by at least three independent experiments performed in triplicate. (**B**–**E**) Activity of KDM5B (B), KDM5C (C), KDM6A (D) and KDM6B (E) with the indicated compounds in AlphaScreen assays. Bars in B-D indicate mean ± SEM.

Our screen identified several known JmjC demethylase inhibitors, as well as new inhibitor chemotypes. For instance, 2-4(4-methylphenyl)-1, 2-benzisothiazol-3(2H)-one (PBIT), 2, 4-pyridinedicarboxylic acid (2, 4-PDCA), caffeic acid, and catechols like methyldopa, carbidopa and levodopa were among the active hits in the screen, validating the capability of our screening methods to identify inhibitors of KDM5A. Among the top hits, we identified several 3-thio-1,2,4-triazole compounds (Table 1, Figure 2B). For further characterization, we focused on the top two inhibitors: YUKA1 (4-([2-(allyloxy)-3-methoxybenzyl]amino)-4H-1,2,4-triazole-3-thiol) and YUKA2 (N-[(4-allyl-5-mercapto-4H-1,2,4-triazol-3-yl)methyl]-3-methyl benzamide), standing for *Y*ale *U*niversity *K*DM5*A* inhibitors *1* and *2*. These inhibitors have average *in vitro* IC_50_ values of 2.66 and 1.32 μM, respectively (Figure 2C, 2D). Interestingly, YUKA1 and YUKA2 showed no activity against KDM5A's close homologue KDM5B at 50 μM (Figure 3A, 3B) and are ~3 and 5 fold less active against KDM5C, respectively (Figure 3A, 3C). Furthermore, 50 μM YUKA1 and YUKA2 did not half-maximally inhibit H3K27 demethylases KDM6A (UTX) and KDM6B (JMJD3) (Figure 3A, 3D, 3E). Therefore, YUKA1 and YUKA2 appear to be specific inhibitors of KDM5A and KDM5C.

**Table 1:** Selection of screen hits with a 3-thio-1,2,4-triazole core

#	Name	Supplier ID	Structure
1	YUKA1 4-([2-(allyloxy)-3-methoxybenzyl]amino)- 4H-1,2,4-triazole-3-thiol	ChemBridge 7870547	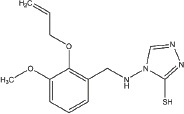
2	YUKA2 N-[(4-allyl-5-mercapto-4H-1,2,4-triazol- 3-yl)methyl]-3-methylbenzamide	ChemBridge 7840569	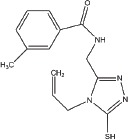
3	4-[(4-methoxybenzyl)amino]- 5-methyl-4H-1,2,4-triazole-3-thiol	ChemBridge 7985526	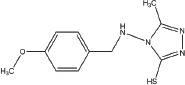
4	5-[2-(butylthio)ethyl]-4-phenyl-4H- 1,2,4-triazole-3-thiol	ChemBridge 7918866	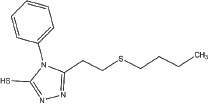
5	5-(4-chlorophenyl)-4-isopropyl-2,4-dihydro -3H-1,2,4-triazole-3-thione	ChemBridge 7809088	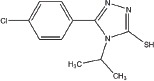
6	2-[(4-methyl-5-phenyl- 4H-1,2,4-triazol- 3-yl)thio]-1-phenylethanone	ChemBridge 7521464	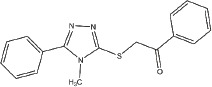

Numbers correspond to the compounds shown in Figure 2.

**Figure 4:**
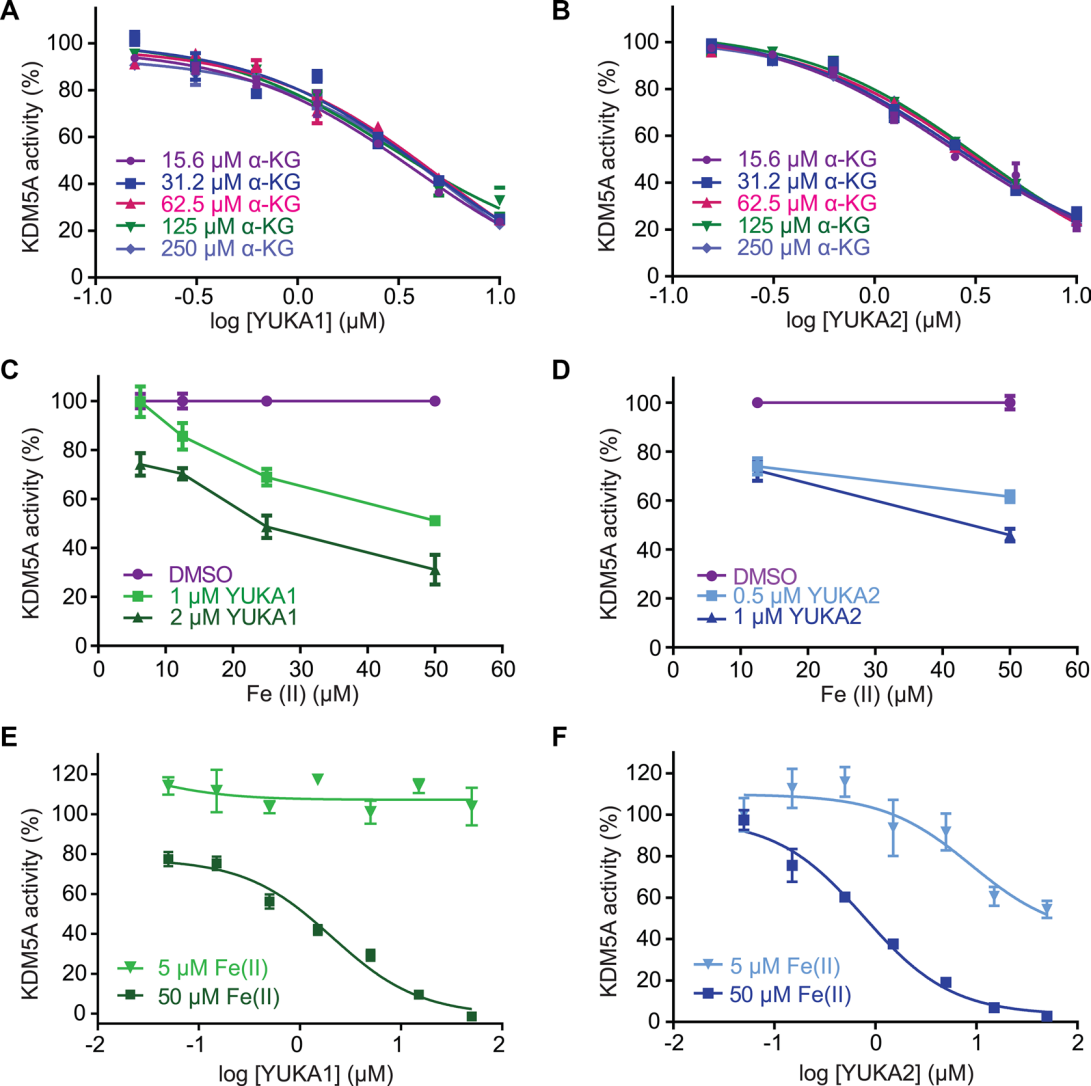
Mechanistic characterization of YUKA1 and YUKA2 (**A**–**B**) Dose-response analysis of YUKA1 (A) and YUKA2 (B) over a 16-fold range of concentrations of α-KG. (**C**–**D**) Inhibition of KDM5A by YUKA1 (C) and YUKA2 (D) in reactions with varying concentrations of Fe(II). (**E**–**F**) Dose-response analysis of YUKA1 (E) and YUKA2 (F) at the indicated Fe(II) concentrations. Data points in A-F indicate mean ± SEM. Data are representative of at least two independent experiments performed in triplicate.

The specificity of these inhibitors prompted investigation of their mechanism of inhibition. Most characterized inhibitors of JmjC demethylases compete with cofactors required for the demethylase reaction, so we performed competition analyses with these cofactors. The IC_50_ values for YUKA1 and YUKA2 did not change significantly over a wide range (16-fold) of concentrations of α-KG, implying that competition with α-KG is not the main mechanism of action (Figure 4A, 4B). These experiments were conducted using concentrations of peptide and Fe(II) several fold greater than their apparent K_m_ values in order to focus on the effect of α-KG alone. Interestingly, analyzing the activity of FLAG-KDM5A at a range of Fe(II) concentrations revealed that Fe(II) is necessary for effective inhibition by YUKA1 and YUKA2 (Figure 4C, 4D). For example, inhibition of KDM5A activity by 2 μM YUKA1 ranged from ~25% at low Fe(II) to ~70% at 50 μM Fe(II). At low Fe(II) concentration (5 μM), enzyme inhibition was too weak to generate robust IC_50_ curves for YUKA1 and YUKA2, as was done easily at 50 μM Fe(II) (Figure 4E, 4F). These results suggest that YUKA1 and YUKA2 are uncompetitive with respect to Fe(II), requiring enzyme-bound Fe(II) for inhibition.

### KDM5A inhibitor YUKA1 inhibited cancer cell proliferation and drug resistance

**Figure 5:**
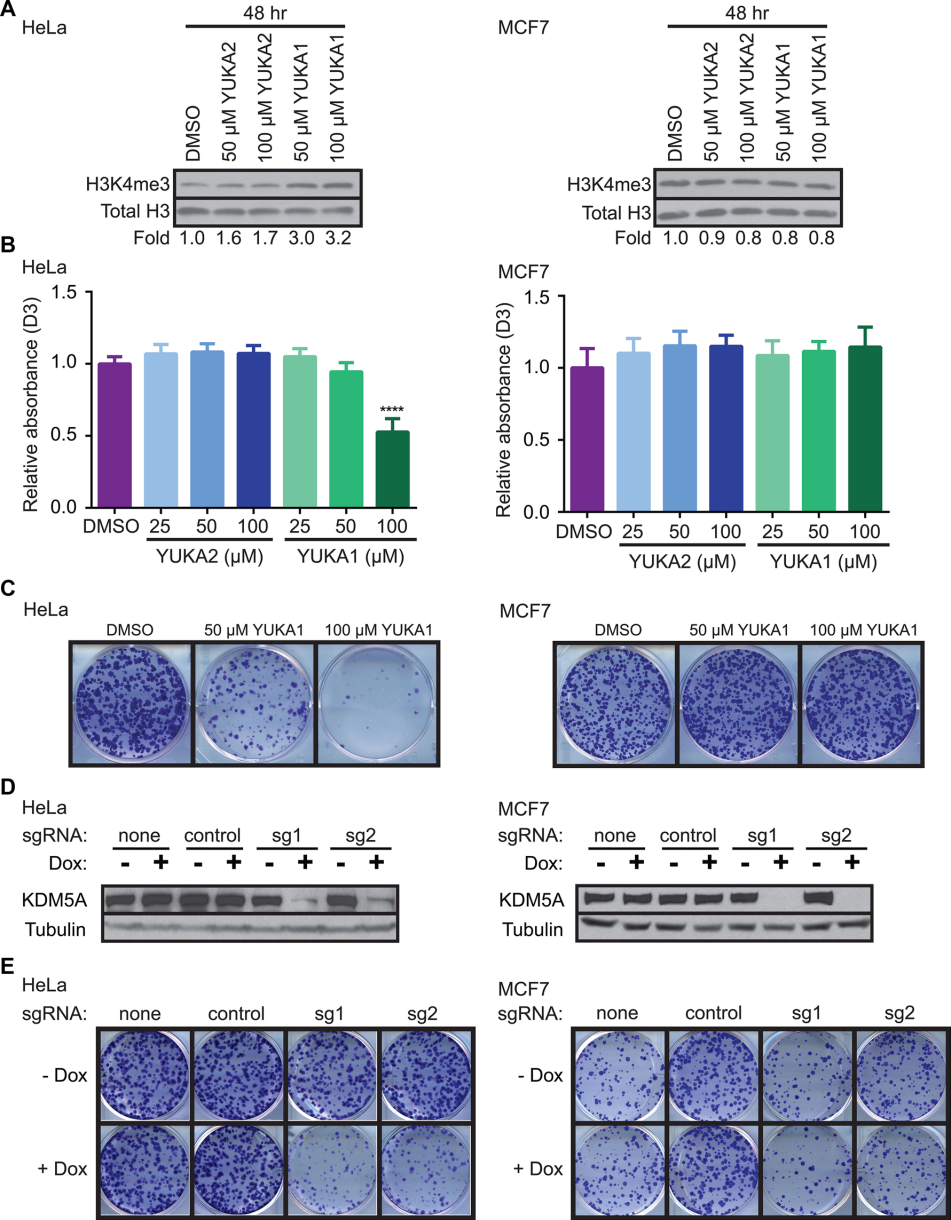
YUKA1 is cell-active and selectively inhibits proliferation of KDM5A-dependent cancer cells (**A**) Representative western blot analysis of H3K4me3 in HeLa (left) and MCF7 (right) cells after 48 hour treatment with the indicated compounds. Fold represents the relative ratio of band intensity for H3K4me3 divided by Total H3 loading control, normalized to DMSO control. (**B**) WST-1 proliferation assays of HeLa (left) and MCF7 (right) cells in the presence of YUKA1 and YUKA2 at the indicated concentrations. Bars indicate mean ± SD of three independent experiments performed in quadruplicate. Asterisks indicate significance by unpaired *t* test (****p < 0.0001). D3, day 3; D0, day 0. (**C**) Colony formation assays of HeLa (left) and MCF7 (right) cells treated with DMSO or YUKA1 for 12 days. Representative wells are shown. Quantification is shown in Supplementary Figure 2A. (**D**) Representative western blot analysis of HeLa (left) and MCF7 (right) cells with doxycycline-induced KDM5A deletion using the CRISPR/Cas9 system. (**E**) Colony formation assays of HeLa (left) and MCF7 (right) cells shown in panel D at 12 (HeLa) or 19 (MCF7) days after induction. Representative wells are shown. Quantification is shown in Supplementary Figure 2B. Dox, doxycycline; sg1, sgRNA 1; sg2, sgRNA 2.

YUKA1 and YUKA2 were tested for their abilities to inhibit KDM5A *in vivo* using HeLa cervical cancer and MCF7 breast cancer cell lines. Western blot analysis of global H3K4 methylation changes revealed that YUKA1, but not YUKA2, was cell-active (Figure 5A). These results are consistent with the fact that YUKA2 possesses a polar amide bond, which likely hinders its permeability across the cell membrane. A dose-dependent increase in global H3K4me3 levels was observed after 48 hour treatment with YUKA1 in HeLa cells, but not in MCF7 cells (Figure 5A). H3K4me2 and H3K4me1 levels were also increased in YUKA1-treated HeLa cells, but not MCF7 cells (Supplementary Figure 1A). The ability of YUKA1 to change global H3K4 methylation levels correlated with its ability to affect the rate of cell proliferation. Proliferation of HeLa cells treated with YUKA1 was less than half of DMSO-treated cells after 3 days, while MCF7 cells were not affected (Figure 5B). Likewise, the number of colonies formed by HeLa cells after a two-week treatment with YUKA1 was significantly reduced compared to treatment with DMSO control, but MCF7 cells treated with YUKA1 formed a similar number of colonies as the control (Figure 5C, Supplementary Figure 2A). To confirm the differential effects of KDM5A inhibition in these two cell lines, we generated HeLa and MCF7 cells with doxycycline-inducible Cas9-mediated knockout of KDM5A (Figure 5D) and examined the effects of KDM5A loss on colony formation. Consistent with YUKA1 inhibition, KDM5A loss significantly decreased the ability of HeLa cells to form colonies, but had little effect on MCF7 cells, as shown by comparing the doxycycline-treated wells to the untreated control wells (Figure 5E, Supplementary Figure 2B).

In order to further validate the cellular function of YUKA1, we tested YUKA1 in ZR-75-1 breast cancer cells, a cell line with KDM5A amplification and in which RNAi-mediated knockdown of KDM5A resulted in decreased cell proliferation [15]. We found that 48 hour treatment with YUKA1 increased global H3K4me3 levels in this cell line (Supplementary Figure 1A), as well as decreased cell proliferation during 5 days of treatment in a dose-dependent manner (Supplementary Figure 1B). The triple negative breast cancer cell line MDA-MB-231 showed only minor changes in H3K4 methylation, accompanied by a small decrease (10–15%) in cell proliferation during 5 days of treatment (Supplementary Figure 1A, 1C). In comparison, treatment with YUKA1 did not affect H3K4 methylation levels and did not hinder cell proliferation of the normal-like MCF10A immortalized mammary epithelial cells (Supplementary Figure 1A, 1D). This corresponds to previous data showing that knocking down KDM5A in MCF10A cells did not reduce cell growth [15].

**Figure 6:**
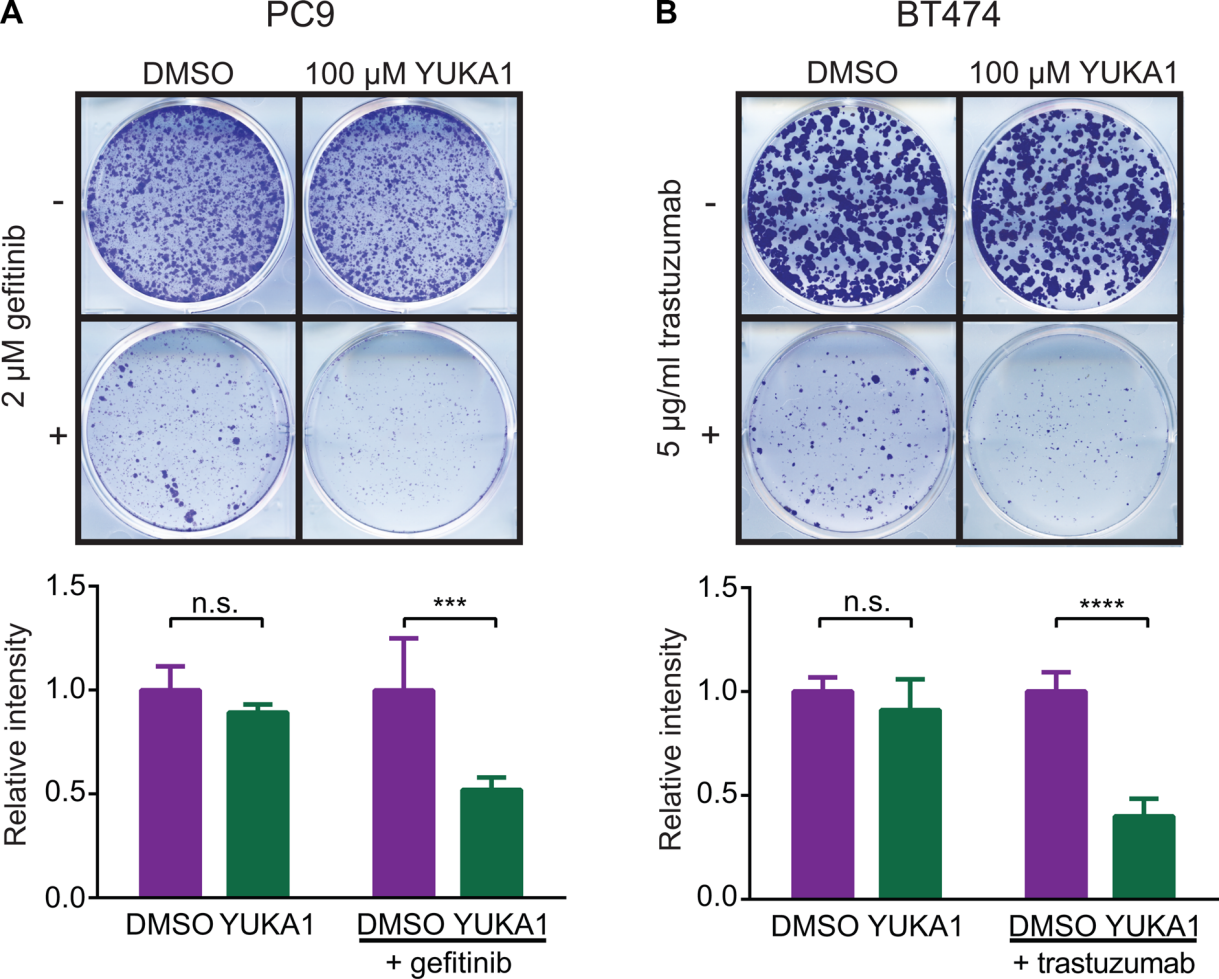
Effect of YUKA1 on anti-cancer drug resistance (**A**) Colony formation assays of PC9 cells treated with DMSO or YUKA1 for 7 days (top wells), or treated with 2 μM gefitinib plus DMSO or YUKA1 for 35 days (bottom wells). (**B**) Colony formation assays of BT474 cells treated with DMSO or YUKA1 for 35 days (top wells), or treated with 5 μg/mL trastuzumab plus DMSO or YUKA1 for 42 days (bottom wells). Representative wells are shown in the top panel and quantification from three independent experiments performed in triplicate is shown in the bottom panel. Asterisks indicate significance by unpaired *t* test (***p = 0.0002; ****p < 0.0001). Bars indicate mean ± SD. Relative intensity is the measured intensity value divided by the average value of DMSO-treated wells.

KDM5A was shown to be a powerful mediator of drug tolerance to gefitinib, a small molecule inhibitor of the epidermal growth factor receptor (EGFR), in the EGFR-mutant lung cancer cell line PC9 [23]. However, it was not known whether the demethylase activity of KDM5A actively contributed to this phenotype. Colony formation assays showed that fewer YUKA1-treated cells formed colonies during long-term treatment with 2 μM gefitinib compared to control cells treated with DMSO (Figure 6A). Growth of PC9 cells was not significantly affected by treatment with YUKA1 alone, which corresponded to prior data showing that KDM5A knockdown did not affect short term proliferation of PC9 cells [23]. We then looked at the effects of YUKA1 in a different setting of anti-cancer drug resistance using human epidermal growth factor receptor-positive (HER2+) BT474 breast cancer cells treated with the monoclonal antibody trastuzumab (trade name Herceptin). BT474 cell growth was not changed when treated with YUKA1 alone, but emergence of colonies subjected to a low dose of trastuzumab (5 μg/ml) was significantly less for cells treated with YUKA1 (Figure 6B). These experiments provide the first evidence that the demethylase activity of KDM5A is necessary for both lung and breast cancer cells to develop resistance to targeted therapies.

## DISCUSSION

This study presents the first screen for potent and specific inhibitors of KDM5A, and to our knowledge, reports the first small molecule inhibitors that are specific for KDM5A over its closely related family member KDM5B. We performed a screen using the full-length KDM5A protein, an approach which enables identification of new inhibitor chemotypes that may not be discovered by screening against truncated KDM5A or by structure-guided design. The screen was performed using the AlphaScreen platform, which is cost-effective, highly sensitive, and requires only small amounts of enzyme (for review of demethylase assays see [32]).

Among ~9,000 compounds screened, we identified 34 compounds that inhibited KDM5A with IC_50_ values in the low μM range (< 5 μM). Several compounds were previously shown as inhibitors of JmjC demethylases, validating the results from our screen. Our screen revealed a novel inhibitor chemotype that includes a core structure of 3-thio-1,2,4-triazole (Table 1). Inhibitors of KDM5A reported in the literature thus far are pan-KDM5 demethylase inhibitors, with strong inhibitory effects on the other KDM5 family members or they were not evaluated for specificity within the KDM5 family. We showed that YUKA1 and YUKA2 are potent and specific inhibitors of KDM5A. They showed little to no activity in biochemical assays against KDM5B, KDM6A and KDM6B even at the highest tested concentration of 50 μM and were less potent against KDM5C (Figure 3). This specificity was also demonstrated in cell-based assays. HeLa and ZR-75-1 cells depended on KDM5A expression for their proliferation (Figure 5E, [15]). The cell permeable compound YUKA1 increased H3K4me3 levels and inhibited growth of HeLa and ZR-75-1 cells (Figure 5 and Supplementary Figure 1). In contrast, MCF7 cells, which were shown to be sensitive to KDM5B down-regulation [33, 34], were not significantly affected by KDM5A deletion or inhibition by YUKA1 (Figure 5). Likewise, the normal-like MCF10A cells and PC9 lung cancer cells were not affected by KDM5A knockdown or by treatment with YUKA1 ([15], [23], Supplementary Figure 1D, Figure 6A). To determine whether the expression levels of KDM5s correlated with YUKA1 sensitivity, we examined the protein levels of KDM5A, B, and C in all of the cell lines we tested (Supplementary Figure 3). KDM5D was not examined because *KDM5D* is located on the Y chromosome and therefore is not expressed in the breast cancer cell lines derived from female patients. Though YUKA1-sensitive HeLa and ZR-75-1 cells expressed relatively higher levels of KDM5A compared to other cell lines, sensitivity to YUKA1 did not strictly correlate with expression levels of KDM5A, B, or C. Instead, sensitivity appears to correlate with the ability of YUKA1 to influence H3K4 methylation in the cell lines.

There are currently no chemical tools to study the demethylase activity of KDM5A separately from that of its family member KDM5B. YUKA1 and YUKA2 are unique in that they inhibit KDM5A with much greater potency than KDM5B. This feature of these compounds makes them useful tools for exploring of the biology of KDM5 enzymes. Importantly, YUKA1 is cell-active and can serve as a way to ascertain the significance of KDM5A's demethylase activity in cells.

YUKA1 and YUKA2 appear to inhibit KDM5A via a novel mechanism of action. As opposed to the α-KG analogues and iron chelators found to inhibit other JmjC demethylases, these inhibitors do not appear to compete with α-KG and require Fe(II) for effective inhibition (Figure 4). Since thiols are known to have a high affinity for iron, it is likely that these compounds bind iron at an open coordination site or possibly displace one or more of iron's natural ligands, thereby disrupting the catalytic cycle.

A crystal structure of truncated KDM5A was recently solved [35], and may aid us in determining the basis for selective inhibition of KDM5A over KDM5B. We recognize that the core structure of the compounds presented here is related to a structure reported to be a pan-assay interference compound [36]. However, YUKA1 and YUKA2 displayed remarkable specificity against KDM5A's closest relatives in the experiments presented here and they proved to be extremely useful tool compounds. Furthermore, catalase, a hydrogen peroxide scavenger, did not affect the inhibitory potency of YUKA1 and YUKA2 against KDM5A, indicating that hydroxyl radicals formed by potential Fenton chemical reactions are not inactivating the protein (Supplementary Figure 4). Efforts to further characterize the mode of action of these compounds are ongoing.

KDM5A has been implicated in cancer processes including cell proliferation [14, 15], metastasis [22] and drug resistance [15, 23, 24]. The tool compounds described here allowed determination of the necessity of KDM5A's catalytic activity in two of those settings. Consistent with previous data from our laboratory and others that KDM5A-dependent cells require its demethylase activity for cell proliferation [14, 18], YUKA1 inhibited proliferation of HeLa cervical cancer cells and ZR-75-1 breast cancer cells (Figure 5B, Supplementary Figure 1B). Moreover, evidence presented here suggests that the demethylase activity of KDM5A is indeed required for the development of drug tolerance to two different targeted therapies, a small molecule and a monoclonal antibody targeting members of the epidermal growth factor receptor family in lung and breast cancer, respectively (Figure 6). This data provides rationale to consider use of KDM5A inhibitors to sensitize cells to established anti-cancer therapy regimens. Taken together, this screen identified specific inhibitors that can be used to study the biology of the KDM5A demethylase.

## MATERIALS AND METHODS

### Histone peptides and antibodies

Biotinylated peptides were purchased from AnaSpec. Peptide sequences were described previously [25]. Anti-H3K4me1 (ab8895), anti-histone H3 (ab1791), and anti-GAPDH (ab9385) polyclonal antibodies were purchased from Abcam. Anti-H3K4me3 (CS9751), anti-H3K4me2 (CS9725) and anti-KDM5A (CS3876) monoclonal antibodies were purchased from Cell Signaling. Anti-H3K27me2 (07–452) polyclonal antibody was purchased from EMD Millipore. Anti-tubulin (T5168) and anti-vinculin (V9131) monoclonal as well as anti-KDM5B (HPA027179) polyclonal antibodies were purchased from Sigma. Anti-KDM5C polyclonal antibody (A301-035A) was purchased from Bethyl Laboratories, Inc. Anti-KDM5A antibody (Kaelin 1416) used in Figures 5D and S3 was described previously [6].

### Cell lines

Sf21 insect cells were cultured at 27°C in Grace's medium (Gibco) with 10% fetal bovine serum (FBS) and 1% penicillin/ streptomycin. HeLa cells were cultured in DMEM (Gibco) with 10% FBS and 1% penicillin/ streptomycin. BT474, MCF7, MDA-MB-231, PC9 and ZR-75-1 cells were cultured in RPMI 1640 (Gibco) with 10% FBS and 1% penicillin/ streptomycin. MCF10A cells were cultured as described previously [25]. All human cells were cultured at 37°C and 5% carbon dioxide. HeLa and MCF7 cell lines were authenticated using short tandem repeat profiling performed at the DNA Analysis Facility on Science Hill at Yale University. All other human cell lines used were within 10 passages after being obtained from the American Type Culture Collection.

### Enzyme production

Sf21 insect cells were infected with baculovirus to express full-length FLAG-KDM5A [6]. After three days at 27°C, cells were harvested and the enzyme was isolated using anti-FLAG M2 beads (Sigma). Samples were run on 7% SDS-PAGE gels, stained with Coomassie Brilliant Blue or used for western blot analysis to verify purity. Full-length FLAG-KDM5B, FLAG-KDM5C, and His-FLAG-KDM6A production was detailed previously [25]. FLAG-KDM6B (1043-1682) was purchased from BPS Bioscience (50115).

### Western blot analysis

Cells were lysed as described previously [14]. Histones were separated by centrifugation, resuspended in Laemmeli buffer, and sonicated. Protein concentration of cell lysates was measured by Bradford assay. Samples in Laemmeli buffer were boiled 10 minutes at 95°C and loaded onto 7% (whole cell lysates) or 18% (histones) SDS-PAGE gels. Membranes were blocked in 4% non-fat milk in Tris-buffered saline with 0.05% Tween (TBS-T) and incubated with primary antibodies in the same buffer or 5% bovine serum albumin in TBS-T overnight at 4°C. Membranes were incubated with secondary anti-rabbit or anti-mouse antibodies for one hour at room temperature. Blots were visualized by Thermo Scientific Pierce ECL Western Blotting Substrate (32106) or EMD Millipore Immobilon Western Chemiluminescent HRP Substrate (WBKLS0100) on film. Signal was quantified using ImageJ software.

### Chemicals

YUKA1 (7870547), YUKA2 (7840569), and other compounds listed in Table 1 were purchased from ChemBridge Hit2Lead. DMSO (9224–01) and sodium chloride (3624–07) were purchased from J.T. Baker. HEPES 1M buffer solution pH 7.3 (AB060201) and Coomassie Brilliant Blue G-250 (AB00325) were purchased from American BioAnalytical. L-ascorbic acid (4407–02) was purchased from Mallinckrodt Chemicals and 2-ketoglutaratic acid (K0005) was purchased from TCI America. Ferrous ammonium sulfate hexahydrate ACS reagent grade (152523) was purchased from MP Biomedicals. Zinc chloride (A16281) was purchased from Alfa Aesar. Trastuzumab (Herceptin) was purchased from Genentech. Gefitinib was purchased from Cayman Chemical Company (13166). Ponceau S was purchased from Acros (161470100).

### Demethylation reactions

Histone demethylase assays were performed as described previously [25], with the exception of all enzyme reactions containing 125 μM α-KG. Enzymes were used at the following approximate concentrations in validation experiments, chosen based on their activity in AlphaScreen assays: 19 nM FLAG-KDM5A, 15 nM FLAG-KDM5B, 6 nM FLAG-KDM5C, 29 nM His-FLAG-KDM6A, and 16 nM FLAG-KDM6B. 24 nM FLAG-KDM5A was used for K_m_ determination experiments. Reactions were carried out for 1 hour, or were stopped by addition of 30 mM EDTA every 5 minutes for determining enzyme kinetics. K_m_ values were calculated using the Michaelis-Menten non-linear regression analysis on GraphPad Prism 6.0 software. The concentration of DMSO in demethylation reactions was 0.05%. For reactions with catalase, catalase (Sigma C30) or an equal volume of dilution buffer was added to the peptide-containing buffer before addition of the demethylase. The final concentration of catalase in the reactions was 0.01 mg/ml.

### AlphaScreen assays

The AlphaScreen general IgG (protein A) detection kit from PerkinElmer Life Sciences was used as described previously [25]. The luminescence emission was recorded at 570 nm using the AlphaScreen optic module on a Pherastar (BMG Labtech) or Envision (PerkinElmer Life Sciences) microplate reader.

### Screen

8,861 compounds from the ChemBridge MW-Set, ChemBridge DIVERSet, MicroSource Gen-Plus, MicroSource Pure Natural Products, NIH Clinical Collection, and Enzo Epigenetics libraries were screened for inhibition of FLAG-KDM5A demethylase activity. Compounds were dissolved in DMSO and added at 20 μM to 384-well white plates (Corning 3574) containing 64 nM bio-H3K4me3 peptide in demethylase buffer prior to addition of 13 nM FLAG-KDM5A. Hits were selected at a threshold of three standard deviations (~30% inhibition). A counter-screen was performed with the hits to eliminate any non-specific compounds. For this, 20 μM compounds were added to 64 nM bio-H3K4me2 peptide in demethylase buffer with no enzyme. Any hits that interfered with the positive signal detection were eliminated. Dose-response curves were performed on selected hits using 0.1–11 μM of compound. Further validation of top hits was performed using 5 μM of each compound or a dose-response curve using 0.05–50 μM of the compound.

### Characterization of top compounds

Fresh compounds were ordered to confirm identity. IC_50_ curves were generated for YUKA1 and YUKA2 using 0.05–50 μM compound in 1 hour reactions. IC_50_ values were calculated by log transformation and non-linear regression log(inhibitor) versus response(three parameters) using Graphpad Prism 6.0 software. Percent activity of KDM5A was calculated for each data point by subtracting the background (average H3K4me3 signal) and dividing by the average signal for the DMSO controls. α-KG competition tests were performed using 150 nM H3K4me3 peptide and 50 μM Fe(II).

### Cell proliferation assays

WST-1 reagent from Roche Applied Sciences (11644807001) was used to measure cell number before drug addition and after 3 or 5 days of growth as described previously [25]. Cells were seeded in 96-well plates at the following number of cells per well: HeLa (2000), MCF7 (2000), ZR-75-1 (1000), MCF10A (1000), MDA-MB-231(5000). DMSO was used at 0.1%. Relative absorbance was calculated by subtracting the average background (media only) signal, dividing by the average signal on day 0, and then dividing by the average day 5/day 0 ratio for the DMSO controls. Significance was calculated by unpaired, two-tailed student's *t*-test using GraphPad Prism 6.0 software.

### Knockout of KDM5A in cell lines

HeLa and MCF7 cells were infected with a lentiviral doxycycline-inducible Cas9-P2A-GFP construct. Cells highly expressing GFP after 2 days of 1 μg/ml doxycycline treatment were selected by flow cytometry and seeded one cell per well in a 96-well plate. Colonies with good GFP induction were harvested from this plate and infected with a lentiviral construct carrying sgRNA lacking a targeting sequence (none), control sgRNA (GACCGGAACGATCTCGCGTA), or one of two sgRNAs targeting KDM5A (sg1: CGTCTTTGAGCCGAGTTGGG, sg2: GATTTCCGGTGAAGGATGGG). Cells were selected by treatment with puromycin (1 μg/ml) for one week and continually cultured in puromycin afterwards. Knockout of KDM5A after a 3-day treatment with doxycycline (1 μg/ml) was confirmed by western blot.

### Colony formation assays

Cells were seeded in 6-well plates at low density: HeLa, MCF7, BT474, and PC9 cells at 1000, 2000, 2500, and 2000 cells/well respectively. Media containing the prescribed drugs or 0.1% DMSO was replaced every 3 days. Trastuzumab was used at 5 μg/ml. Gefitinib was used at 2 μM. YUKA1 was used at 50 or 100 μM. Doxycycline was used at 1 μg/ml for 3 days. After 7–42 days depending on the rate of cell growth, cells were fixed in 4% para-formaldehyde in phosphate-buffered saline for 10 minutes rocking at room temperature. They were incubated in 0.05% crystal violet in double-distilled water for 30 minutes rocking at room temperature, washed with water, and dried 24 hours before photographing. ColonyArea, a plugin for ImageJ software, was used for quantification [37]. Unpaired, two-tailed student's *t*-test calculated using GraphPad Prism 6.0 software was used to determine significance.

## SUPPLEMENTARY MATERIALS


