# Supplementary material for: Screen-identified selective inhibitor of lysine demethylase 5A blocks cancer cell growth and drug resistance
Source: Oncotarget. 2016 May 21;7(26):39931–44. doi: 10.18632/oncotarget.9539 (PMC5129982; doi:10.18632/oncotarget.9539)
Supplement: Supplementary file 1 [file oncotarget-07-39931-s001.pdf]

Screen-identified selective inhibitor of lysine demethylase 5A blocks cancer cell growth and drug resistance

Supplementary Materials

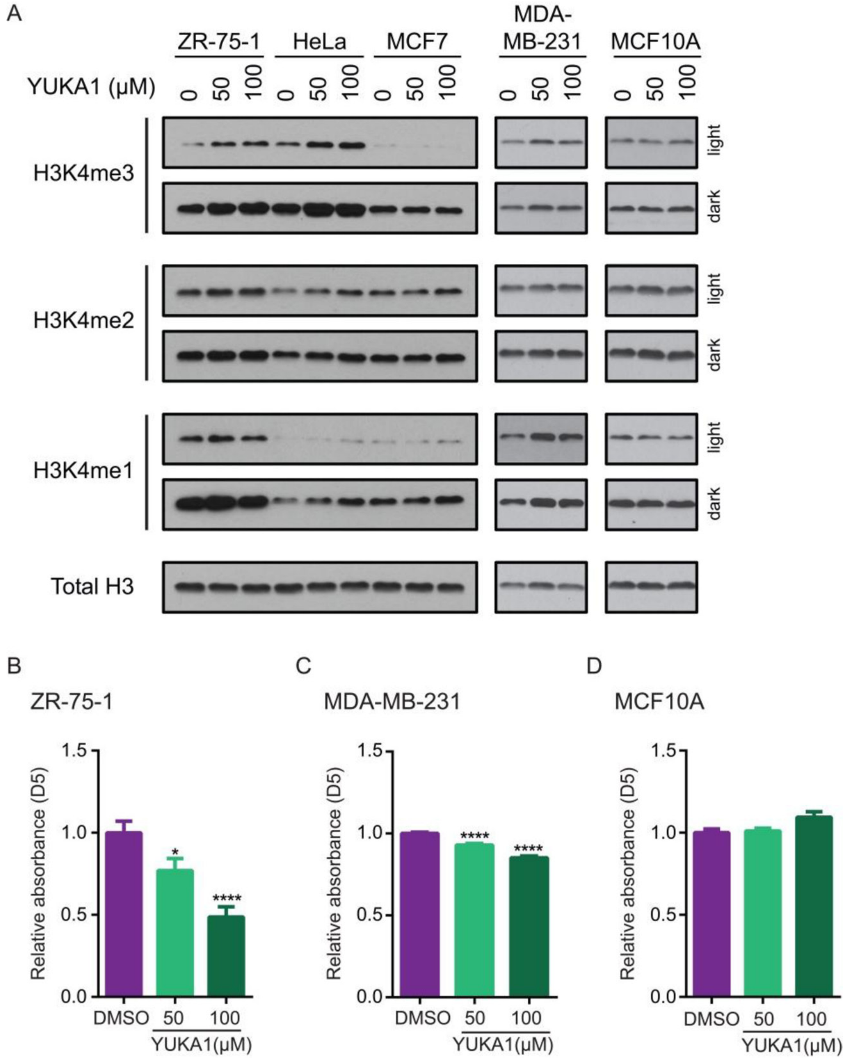

Supplementary Figure S1: Effect of YUKA1 on H3K4 methylation and proliferation of normal-like and cancer cells. (**A**) Representative western blot analysis of H3K4 methylation in ZR-75-1, HeLa, and MCF7 cells after 48 hr treatment with YUKA1, as well as MDA-MB-231 and MCF10A cells after 72 hr treatment with YUKA1. Total H3 serves as the loading control. Two exposures (light and dark) are shown for each blot. (**B**) WST-1 proliferation assays of ZR-75-1 cells treated with YUKA1 for 5 days. Bars indicate mean ± SEM of four independent experiments performed in quadruplicate. (**C**) WST-1 proliferation assays of MDA-MB-231 cells treated with YUKA1 for 5 days. Bars indicate mean ± SEM of three independent experiments performed in quadruplicate. (**D**) WST-1 proliferation assays of MCF10A cells treated with YUKA1 for 5 days. Bars indicate mean ± SEM of three independent experiments performed in quadruplicate. In B-D, asterisks indicate significance by unpaired *t* test (**p* = 0.03; *****p* < 0.0001). D5, day 5.

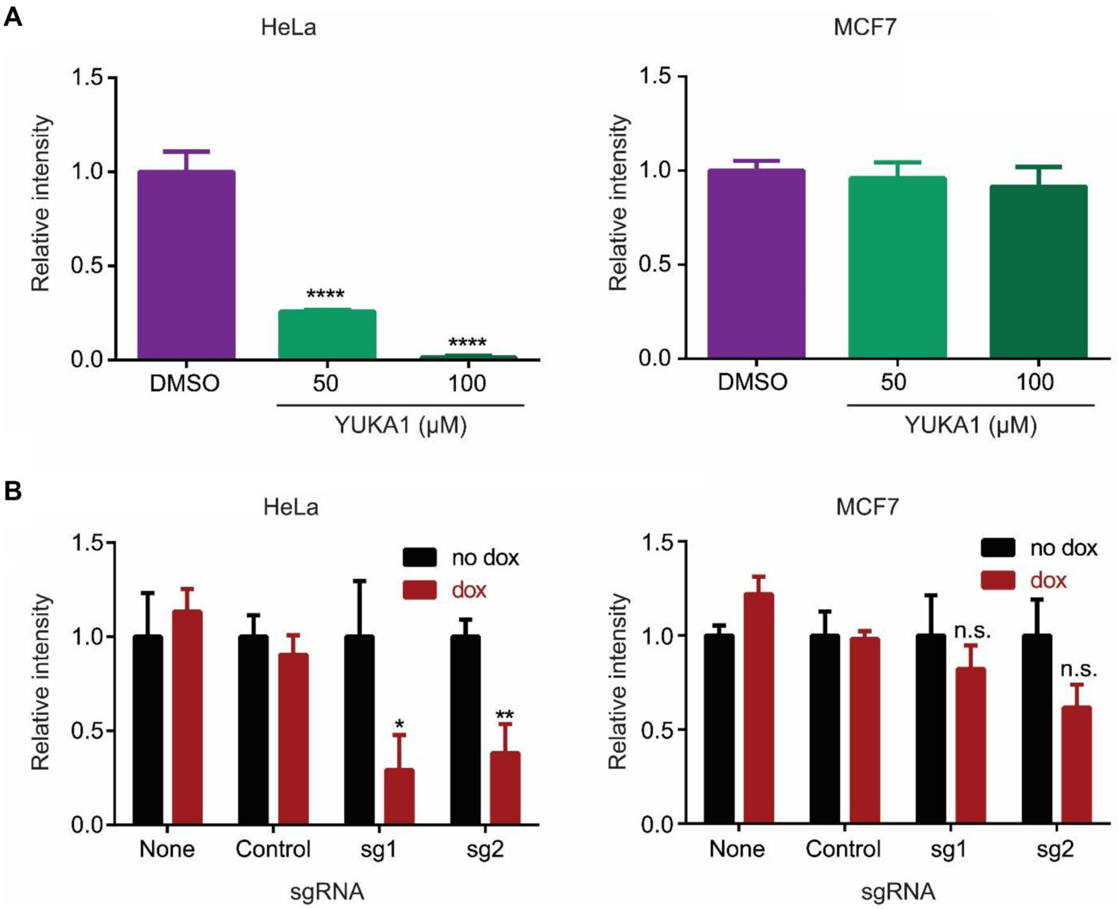

Supplementary Figure S2: Quantification of colony formation assays shown in Figure 5. (**A**) Quantification of HeLa (left) and MCF7 (right) colony formation assays represented in Figure 5C. Bars indicate mean ± SD for two independent experiments in triplicate. Asterisks indicate significance by unpaired *t* test (****$p < 0.0001$). (**B**) Quantification of HeLa (left) and MCF7 (right) cells represented in Figure 5E. Bars indicate mean ± SD. dox, doxycycline. Asterisks indicate significance by unpaired *t* test (*$p = 0.02$; **$p = 0.004$; NS, $p > 0.05$). Relative intensity in A–B is the measured intensity value divided by the average value of DMSO-treated wells.

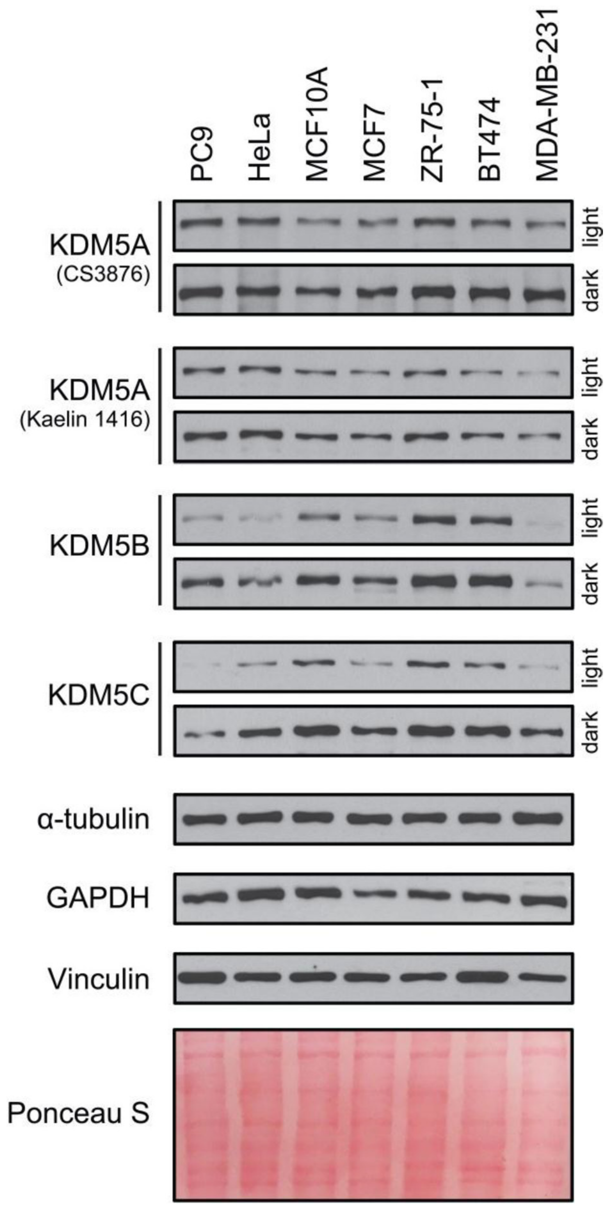

Supplementary Figure S3: Expression of KDM5 demethylases in cell lines. Representative Western blot analysis of KDM5A (two different antibodies), KDM5B, and KDM5C in lung cancer cells (PC9), cervical cancer cells (HeLa), immortalized mammary epithelial cells (MCF10A), and breast cancer cell lines (MCF7, ZR-75-1, BT474, MDA-MB-231). Two exposures (light and dark) are shown for KDM5 blots. As expression of housekeeping loading control proteins varies across cell lines of different tissue types, equal loading was assessed by α-tubulin, GAPDH, vinculin, as well as Ponceau S membrane staining.

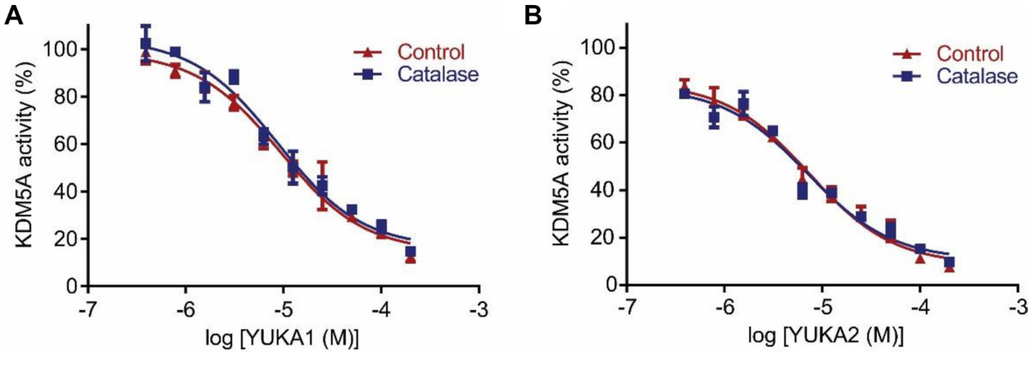

Supplementary Figure S4: Catalase does not obstruct inhibition by YUKA1 and YUKA2. AlphaScreen assays assessing KDM5A activity performed in the presence of 0.01 mg/ml catalase or vehicle control reactions for YUKA1 (**A**) and YUKA2 (**B**). Data points and bars in A–B indicate mean ± SD.
